# Natural supplementation to effectively treat cancer-induced fatigue: evidence of a meta-analysis on the use of guaraná

**DOI:** 10.1590/1806-9282.20240528

**Published:** 2024-11-11

**Authors:** Jean Henri Maselli-Schoueri, Pedro Nazareth Aguiar, Auro del Giglio

**Affiliations:** 1 Centro Universitário Faculdade de Medicina do ABC, Department of Oncology – Santo André (SP), Brazil.; 2 Oncoclínicas Group – São Paulo (SP), Brazil.

**Keywords:** Cancer-induced fatigue, Natural supplements, Guaraná, Quality of life, Cancer patients

## Abstract

**BACKGROUND:**

Cancer-related fatigue is a pervasive symptom, affecting up to 90% of cancer patients throughout their illness, and can persist well after treatment has ended. Despite its prevalence, no definitive evidence-based treatment exists, leading to an abundance of proposed alternatives, including natural supplements. To further explore the potential effects of guaraná on cancer-related fatigue, a systematic review with meta-analysis was conducted and registered on the International Prospective Register of Systematic Reviews (CRD42023484144).

**METHODS:**

A thorough search of PubMed/MEDLINE and Cochrane Library was conducted using MeSH terms and related keywords for cancer-related fatigue and guaraná. Eligible studies were selected according to predetermined criteria and assessed for quality in accordance with the Cochrane Handbook for Systematic Reviews of Interventions. Data extraction was independently performed by two reviewers, with any discrepancies resolved by a third researcher.

**RESULTS:**

In total, 4 full articles and 1 abstract, encompassing a total of 229 patients from 2009 to 2023, were included in the meta-analysis. Despite high heterogeneity between studies (I^2^=78%, ꭓ^2^=18.51, df=4, p=0.0010), the analysis revealed a significant benefit of using guaraná to alleviate cancer-related fatigue, with a standard mean difference of −0.77 (95%CI −1.34, −0.21) and a test for the overall effect of Z=2.68 (p=0.007).

**CONCLUSION:**

This meta-analysis provides support for the use of guaraná in the treatment of cancer-related fatigue. However, further investigation through larger prospective randomized controlled trials is necessary to validate these findings.

## INTRODUCTION

Cancer-related fatigue (CRF) is a commonly reported symptom among cancer patients, with estimates suggesting that up to 90% may experience it during their illness, and some may experience it even after cancer treatment is completed^1,2^. The causes of CRF are multifactorial, with systemic inflammation, endocrine, and even psychological factors playing a role^3–7^.

Given the significant impact of CRF on patients’ overall well-being, the search for effective treatments remains an ongoing endeavor^3–7^. While both non-pharmacological and pharmacological interventions have been explored, the latter often have limited evidential support, leading to much debate^5^. In light of this, a growing number of international groups have turned their attention toward natural supplements as a potential adjuvant therapy^8^. In fact, previous studies conducted by the Oncology Department at ABC Medical School examined the use of a purified extract of guaraná (Paullinia cupana), a plant indigenous to the Amazon region of Brazil, and reported promising results in clinical trials with cancer patients undergoing chemotherapy. However, due to the limited number of participants involved, the overall evidential significance of these findings remains low^9–12^.

To address this limitation and consolidate the available evidence for the effects of guaraná on CRF, Araujo et al. conducted a meta-analysis^13^. However, upon review, certain discrepancies were identified, which could potentially be addressed through access to individual patient data. Furthermore, additional evidence regarding the effects of guaraná on CRF has emerged since the initial meta-analysis was conducted^14^, highlighting the need for a new meta-analysis to synthesize the findings from all randomized clinical trials conducted thus far.

## METHODS

The present study followed the standards set by the Preferred Reporting Items for Systematic Reviews and Meta-Analyses (PRISMA) and was registered with the International Prospective Register of Systematic Reviews (PROSPERO) under registration code CRD42023484144. It involved a systematic review conducted between December 1, 2023, and February 1, 2024, with the goal of identifying relevant literature on the effects of guaraná supplementation on CRF. Articles written in English were retrieved from databases such as PUBMED, MEDLINE, LILACS, Embase, Web of Science, FSTA, clinicaltrials.gov, WHO-ICTRP, MedRxiv, and Cochrane Library. The search utilized Medical Subject Headings (MeSH) descriptors including: "Fatigue"[Mesh] OR Fatigue OR Lassitude AND "Paullinia"[Mesh] OR Paullinias OR Paullinia OR (Paullinia cupana) OR (Paullinia cupanas) OR (cupana, Paullinia) OR Guarana OR Guaranas OR (Paullinia pinnata) OR (Paullinia pinnatas) OR (pinnata, Paullinia) OR Barbasco OR Barbascos OR "guarana powder" [Supplementary Concept] OR (guarana powder) OR (guarana supplement). "Fatigue," "Paullinia," "Guarana," and "Guarana powder."

A PICO strategy was implemented to identify relevant studies, with the following criteria:

1. clinical trial,
2. adult population (age ≥18 years),
3. evaluation of the association between guaraná supplementation and CRF, and (4) well-established primary outcome measures such as the use of standard fatigue assessment scales as the BFI.

Additionally, the references to selected articles were screened for eligibility, as well as abstracts from relevant conferences. Two reviewers independently assessed all articles for inclusion, with a third reviewer resolving any discrepancies. Studies were deemed eligible if they met the aforementioned criteria. Studies not reporting outcomes such as the hazard ratio for reduction of fatigue with guaraná compared to placebo were excluded. Data were extracted from eligible studies, including title, authors, publication year and journal, sample size, formulation and active ingredient, medication regimen, and outcomes evaluated. For studies with data available for analysis, a meta-analysis was performed using Review Manager version 5.3.16, with standard deviations (SD) calculated using the RevMan calculator^15^. The risk of bias and assumptions for analysis were assessed in accordance with the Cochrane Handbook for Systematic Reviews of Interventions, employing the GRADE approach^16,17^. The random effects model was utilized, with a significance level of 5%.

## RESULTS

The present review identified a total of 117 articles with MeSH terms related to guaraná and CRF in six different databases. Upon removing duplicates, the remaining 43 articles underwent further screening, of which 11 were deemed relevant based on the established inclusion criteria. After a full abstract review, seven studies met all criteria and were included in the final analysis, consisting of five articles, one abstract, and one PhD thesis^9-11,14,18-20^. Ultimately, a total of 371 patients with cancer diagnoses were included in the review, with the majority of cases being breast cancer. The intervention phase ranged from 90 days or less, with only one study investigating a dose greater than 100 mg of guaraná per day. The Brief Fatigue Inventory (BFI) was the most commonly utilized instrument to measure and analyze fatigue, with a relatively even distribution of positive and negative findings across the included studies. Of note, two articles did not provide outcome data and were therefore excluded from the subsequent meta-analysis. Notably, we excluded two studies by Sette et al. published together in a single paper and the paper by dos Santos Martins et al. also due to insufficient outcome data availability (Table 1)^11,18^.

**Table 1:** Summary of characteristics of studies included in the systematic review.

Author's name and year of publication	Cancer	N*	Intervention	Crossover	Duration of intervention	Instruments for analysis	Outcome
Costa Miranda et al., 2009^20^	Breast	36	Guaraná extract (75 mg) daily	Yes	14 radiotherapy sessions	Chalder Fatigue Scale	Negative
						BFI^a^	
						Beck Inventory Depression	
Oliveira Campos et al., 2011^10^	Breast	60	Guaraná extract (50 mg) 2x a day	Yes	21 days	BFI^a^	Positive
						FACIT-F^b^	
						Chalder Fatigue Scale	
						FACT-ES^c^	
						HADS^d^	
						Pittsburgh Sleep Quality Index	
Giglio et al., 2013^9^	Multiple	33	Guaraná dried extract (PC-18) 37.5 mg 2x a day	Yes	21 days	BFI^a^	Positive
						FACIT-F^b^	
						Chalder	
						HADS^d^	
						PSQI^e^	
Martins et al., 2016^18^	Head and neck	60	Guaraná extract (50 mg) 2x a day	No	42 days	General Questionnaire	Negative
						Guaraná Toxicity Assessment	
						FACT-F^b^	
						FACIT-HN^f^	
						EORTC QLQ-30^g^	
						QLQ H&N35^h^	
Sette et al., 2017^11^	Breast	32	Guaraná dried extract (PC-18) 37.5 mg 2x a day	Yes	21 days	BFI^a^	Negative
						Chalder	
	Breast	72	Guaraná dried extract (PC-18) 7.5 mg or 12.5 mg 2x a day	No	21 days	BFI^a^	Negative
						Chalder	
Albarnaz et al., 2017^19^	Breast	42	Guaraná extract (50 mg) 2x a day	No	90 days	Piper Fatigue Scale	Positive
Inglis et al., 2023^14^	Not informed	36	Guaraná powder (Energy bar) 280 mg or 560 mg per day	No	42 days	BFI^a^	Positive

* Number of patients analyzed in the study.

a Anderson Brief Fatigue Inventory.

b Functional Assessment of Chronic Illness Therapy–Fatigue.

c Functional Assessment of Chronic Illness Therapy–Endocrine Symptoms.

d Hospital Anxiety and Depression Scale.

e Pittsburgh Sleep Quality Index.

f Functional Assessment of Chronic Illness Therapy–Head and Neck Cancer.

g European Organization for Research and Treatment of Cancer Quality of Life Questionnaire Core 30.

h European Organization for Research and Treatment of Cancer Quality of Life Questionnaire-H&N35.

**Figure 1:**
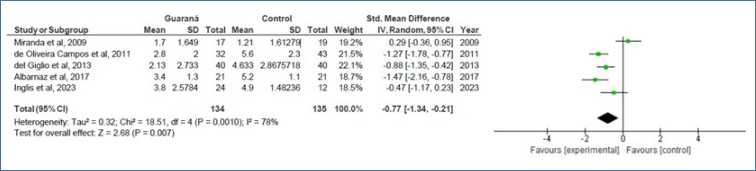
Forest plot of all studies included in the meta-analysis.

Of the five studies selected for meta-analysis, four were published as full manuscripts and one was an abstract containing sufficient data for statistical analysis. Thus, 269 patients were included in the meta-analysis, with some overlap in cases due to crossover study designs. A Cochran's Q test and Higgin and Thompson's I^2^ statistic revealed significant heterogeneity among the included studies, with a Tau^2^ value of 0.32, Chi² value of 18.51 with 4 degrees of freedom (p=0.0010), and an I² value of 78%. The results indicated a statistically significant difference in favor of guaraná for the treatment of CRF, with a mean of −0.77 (95%CI −1.34, −0.21) and a p-value of 0.007 (Figure 1). However, it should be noted that the overall quality of evidence according to the GRADE framework for all studies included was low (Chart 1).

**Chart 1:** Quality of evidence according to GRADE.

Guaraná for fatigue compared to placebo for fatigue						
Patient or population: Fatigue Setting: Intervention: Guaraná for fatigue Comparison: Placebo						
Outcomes	Anticipated absolute effects^*^ (95%CI)		Relative effect (95%CI)	N° of participants (studies)	Certainty of the evidence (GRADE)	Comments
	Risk with placebo	Risk with guaraná for fatigue				
Fatigue	–	SMD 0.77 lower (1.34 lower to 0.21 lower)	–	269 (5 RCTs)	⨁⨁◯◯ Low	
^*^The risk in the intervention group (and its 95% confidence interval) is based on the assumed risk in the comparison group and the relative effect of the intervention (and its 95%CI). CI: confidence interval; SMD: standardized mean difference						
**GRADE Working Group grades of evidence** **High certainty:** We are very confident that the true effect lies close to that of the estimate of the effect. **Moderate certainty:** We are moderately confident in the effect estimate: the true effect is likely to be close to the estimate of the effect, but there is a possibility that it is substantially different. **Low certainty:** Our confidence in the effect estimate is limited: the true effect may be substantially different from the estimate of the effect. **Very low certainty:** We have very little confidence in the effect estimate: the true effect is likely to be substantially different from the estimate of effect.				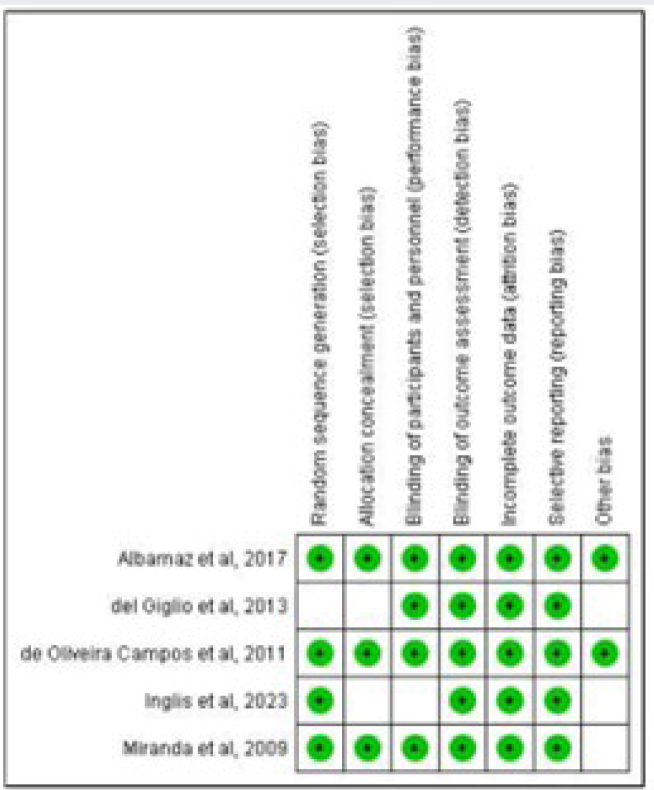		
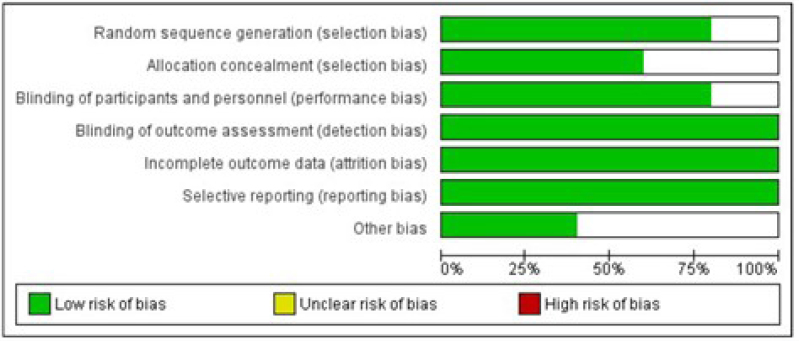						

## DISCUSSION

In this systematic review with meta-analysis, it was determined that the utilization of guaraná may hold promise as a potential intervention for the treatment of CRF. This finding is particularly significant given the current landscape, in which effective treatments for this condition are lacking, and there is a growing interest among both patients and researchers in natural and complementary therapies^21–23^.

It is important to note, however, that our results differ from a previous meta-analysis by Araujo et al.^13^. Upon careful examination, it becomes apparent that this previous meta-analysis contains certain inconsistencies. For instance, the authors have misinterpreted the results when utilizing data from Sette et al., as hazard ratios and SD for the BFI scores comparing placebo with guaraná dry extract PC-18 were unable to be derived from the publication^11^. Furthermore, even when individual patient data were accessible, the researchers were unable to obtain these outcome data. As such, these two studies were excluded from our meta-analysis, and given their negative results, it is possible that their inclusion in the analysis by Araujo et al. may have impacted the overall findings. In addition, when utilizing the dataset of Oliveira Campos et al., the authors specifically opted to present functional assessment of chronic illness therapy–fatigue (FACIT-F) data, as opposed to BFI results, which may have resulted in increased study heterogeneity^10^. It should also be noted that our more recent meta-analysis had access to the data of Ingris et al., provided in abstract form by the University of Rochester team, which had not yet been presented at the time of the de Araujo et al.'s study^11,14^.

From a practical standpoint, our findings hold significant implications, suggesting that physicians managing cancer patients experiencing fatigue may consider incorporating guaraná supplementation as a viable therapeutic option, particularly for patients who are seeking alternative natural treatment modalities or who have found conventional therapies to be ineffective. In this regard, it should be recognized that other non-pharmacological approaches may also have utility, such as exercise and yoga^24,25^.

It must also be acknowledged that no consensus currently exists regarding the appropriate dosage of guaraná for the improvement of CRF. It should also be acknowledged that our study comes with certain limitations, including its reliance on preexisting research data, the small sample sizes of the included studies, and the limitation that most of the participants were from a single country, which may restrict the generalizability of our findings.

In conclusion, our meta-analysis provides promising evidence for the use of guaraná supplementation as an adjunctive strategy for addressing CRF. However, larger-scale prospective randomized controlled trials are warranted to validate our findings.

## References

[1] Hofman M, Ryan JL, Figueroa-Moseley CD, Jean-Pierre P, Morrow GR (2007). Cancer-related fatigue: the scale of the problem. Oncologist.

[2] Thong MSY, Noorden CJF, Steindorf K, Arndt V (2020). Cancer-related fatigue: causes and current treatment options. Curr Treat Options Oncol.

[3] Bower JE (2014). Cancer-related fatigue--mechanisms, risk factors, and treatments. Nat Rev Clin Oncol.

[4] Dagnelie PC, Pijls-Johannesma MC, Lambin P, Beijer S, Ruysscher D, Kempen GI (2007). Impact of fatigue on overall quality of life in lung and breast cancer patients selected for high-dose radiotherapy. Ann Oncol.

[5] National comprehensive cancer network (2023). Cancer-Related Fatigue – version 2.2024.

[6] Visser MR, Smets EM (1998). Fatigue, depression and quality of life in cancer patients: how are they related?. Support Care Cancer.

[7] Charalambous A, Kouta C (2016). Cancer related fatigue and quality of life in patients with advanced prostate cancer undergoing chemotherapy. Biomed Res Int.

[8] Inglis JE, Lin PJ, Kerns SL, Kleckner IR, Kleckner AS, Castillo DA (2019). Nutritional interventions for treating cancer-related fatigue: a qualitative review. Nutr Cancer.

[9] Giglio AB, Cubero Dde I, Lerner TG, Guariento RT, Azevedo RG, Paiva H (2013). Purified dry extract of Paullinia cupana (guaraná) (PC-18) for chemotherapy-related fatigue in patients with solid tumors: an early discontinuation study. J Diet Suppl.

[10] Oliveira Campos MP, Riechelmann R, Martins LC, Hassan BJ, Casa FB, Giglio A (2011). Guarana (Paullinia cupana) improves fatigue in breast cancer patients undergoing systemic chemotherapy. J Altern Complement Med.

[11] Sette CVM, Ribas Alcântara BB, Schoueri JHM, Cruz FM, Cubero DIG, Pianowski LF (2018). Purified dry paullinia cupana (PC-18) extract for chemotherapy-induced fatigue: results of two double-blind randomized clinical trials. J Diet Suppl.

[12] Subbiah MT, Yunker R (2008). Studies on the nature of anti-platelet aggregatory factors in the seeds of the Amazonian Herb Guarana (Paullinia cupana). Int J Vitam Nutr Res.

[13] Araujo DP, Pereira PTVT, Fontes AJC, Marques KDS, Moraes ÉB, Guerra RNM (2021). The use of guarana (Paullinia cupana) as a dietary supplement for fatigue in cancer patients: a systematic review with a meta-analysis. Support Care Cancer.

[14] Abstracts for MASCC/JASCC/ISOO annual meeting 2023 (2023). Support care cancer.

[15] Drahota A, Beller E Cochrane library.

[16] Higgins J, Green S (2017). Cochrane handbook of systematic reviws of interventions 5.2 [Internet].

[17] Carvalho APV, Silva V, Grande AJ (2013). Avaliação do risco de viés de ensaios clínicos randomizados pela ferramenta da colaboração Cochrane. Diagn Tratamento.

[18] Martins SPDS, Ferreira CL, Giglio A (2017). Placebo-controlled, double-blind, randomized study of a dry guarana extract in patients with head and neck tumors undergoing chemoradiotherapy: effects on fatigue and quality of life. J Diet Suppl.

[19] (2007). Albarnaz MDEfetividade do guaraná (paullinia cupana) para manejo da fadiga em mulheres com câncer de mama em quimioterapia: um ensaio clínico, duplo cego, randomizado.

[20] Costa Miranda V, Trufelli DC, Santos J, Campos MP, Nobuo M, Costa Miranda M (2009). Effectiveness of guaraná (Paullinia cupana) for postradiation fatigue and depression: results of a pilot double-blind randomized study. J Altern Complement Med.

[21] Viscuse PV, Price K, Millstine D, Bhagra A, Bauer B, Ruddy KJ (2017). Integrative medicine in cancer survivors. Curr Opin Oncol.

[22] Qureshi M, Zelinski E, Carlson LE (2018). Cancer and complementary therapies: current trends in survivors’ interest and use. Integr Cancer Ther.

[23] Smith WB, Olaku O, Michie J, White JD (2008). Survey of cancer researchers regarding complementary and alternative medicine. J Soc Integr Oncol.

[24] Puetz TW, Herring MP (2012). Differential effects of exercise on cancer-related fatigue during and following treatment: a meta-analysis. Am J Prev Med.

[25] Liu YC, Hung TT, Konara Mudiyanselage SP, Wang CJ, Lin MF (2022). Beneficial exercises for cancer-related fatigue among women with breast cancer: a systematic review and network meta-analysis. Cancers (Basel).

